# Impedance-based cell monitoring: barrier properties and beyond

**DOI:** 10.1186/2045-8118-10-5

**Published:** 2013-01-10

**Authors:** Kathrin Benson, Sandra Cramer, Hans-Joachim Galla

**Affiliations:** 1 Institut für Biochemie, Westfälische Wilhelms-Universität Münster, Wilhelm Klemm Straße 2, Münster, 48149, Germany

**Keywords:** Barrier forming cells, Endothelium, Epithelium, Blood–brain barrier, Electrical resistance, TEER, Impedance spectroscopy

## Abstract

In multicellular organisms epithelial and endothelial cells form selective permeable interfaces between tissue compartments of different chemical compositions. Tight junctions which connect adjacent cells, control the passage of molecules across the barrier and, in addition, facilitate active transport processes. The cellular barriers are not static but can be deliberately modulated by exposure to specific external stimuli. *In vitro* models representing the essential absorption barriers of the body are nowadays available, thus allowing investigation of the parameters that control permeability as well as transport processes across those barriers. Independent of the origin of the barrier forming cells, techniques are needed to quantify their barrier integrity. One simple assay is to measure the permeability for given hydrophilic substrates possessing different molecular weights like sucrose or dextrans. However, this technique is time-consuming and labor-intensive. Moreover, radioactive or fluorescently-labeled substrates are needed to allow easy analytical detection. Finally, if transport processes are investigated, the standard permeant may interfere with the transport process under investigation or might even alter the barrier integrity by itself. Thus, independent, non-invasive techniques are needed to quantify the barrier integrity continuously during the experiment. Such techniques are available and are mainly based on the measurement of the transendothelial or transepithelial electrical resistance (TEER) of barrier forming cells grown on porous membranes. Simple devices using two sets of electrodes (so-called Voltohmeters) are widely used. In addition, an easy-to-use physical technique called impedance spectroscopy allows the continuous analysis of both the TEER and the electrical capacitance giving additional information about the barrier properties of cells grown on permeable membranes. This technique is useful as a quality control for barrier forming cells. Another impedance-based approach requires cells to be grown directly on solid, micro-structured electrodes. Here, we will discuss the physical background of the different techniques; advantages, disadvantages, and applications will be scrutinized. The aim is to give the reader a comprehensive understanding concerning the range and limits of the application, mainly focusing on endothelial cells.

## Review

### Barrier-forming cells

A characteristic feature of epithelial as well as endothelial cell layers is the formation of intercellular junctions resulting in a tight cellular barrier separating the apical (luminal) from the basolateral (abluminal) side. These cell layers form selectively permeable interfaces between compartments of different chemical composition, thus controlling diffusion along the paracellular way as well as transport processes through intracellular pathways. This is guaranteed by the so-called tight junctions (intercellular connections) that seal the intercellular cleft [1]. An intact barrier is crucial for the physiological activities of the corresponding tissue. However, the barrier is not static but can be modulated by specific stimuli to open and close selectively, thus allowing controlled passage from the blood to the brain or *vice versa*[2-4]. Developing methods to overcome the barrier is an important issue and highly relevant for medical treatment of diseases within the barriers. Drug delivery systems or strategies to open the barrier temporarily will help to allow medication to cross the blood–brain and the blood-CSF (cerebrospinal fluid) barrier as well as other barriers such as in the intestine, the kidney, the testis and the placenta. Adequate *in vitro* models are nowadays available, based on cell cultures grown on permeable supports [5]. These are of major importance since the setup offers full access to both the apical and the basolateral compartments. *In vitro* models can, in principle, be based on primary cells [6,7] or cell lines [8,9]. However, in case of blood–brain barrier (BBB) models most cell lines do not express barrier properties similar to *in vivo* conditions [10]. Thus care has to be taken if such cells are used for transport experiments.

Since the presence of an intact barrier is crucial for reliable *in vitro* experiments, techniques had to be developed to quantify the barrier integrity. One possibility is to measure the permeability for small hydrophilic substances like radioactively labeled sucrose or low molecular weight fluorescent dyes [11]. A straightforward method is the measurement of the TEER. A scrutiny of different TEER measurements will be given here. The major focus will be on techniques that allow automated long-term monitoring of barrier-relevant parameters including the dynamic processes within the tight junction network.

### TEER measurement according to Ohm`s law

**Figure 1:**
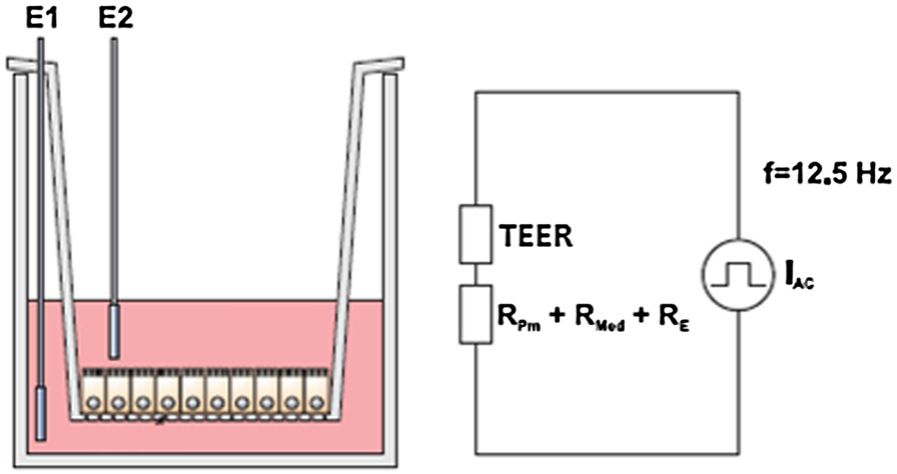
**Impedance measurements with chopstick-like electrodes.** The chopstick-like electrodes (E1,E2) are traditionally used to determine the electric resistance of cells grown on filter inserts. The ohmic resistance of the cell layer (TEER), the cell culture medium in the upper and lower compartment (R_Med_), the membrane of the filter inserts (R_pm_) and electrode-medium interface (R_E_) all contribute to the total electric resistance. I_AC_: alternating current. Adapted from [14] with permission.

A quantitative measure describing the barrier integrity is the electrical, ohmic resistance of the cell layer. In principle it can be determined by a simple, direct current (DC) based approach: a defined DC voltage, U, is applied to two electrodes, one on each side of the cell layer. The resulting current, I, is measured leading to the ohmic resistance R according to Ohm`s law (R = U/I). However the DC current can easily cause adverse effects on both the cells and the electrodes. The latter point has been avoided in case of the widely-used, so-called Epithelial Voltohmmeter (EVOM) [12]. Here an alternating current (AC) square wave with a frequency of 12.5 Hz is used to avoid charging of the cell layer and the electrodes (Figure 1). However, as a handheld device with a chopstick-like arrangement of the electrodes, the resulting TEER readings depend strongly on the position of the probing electrodes. The required movement of the electrodes from one well to the other easily causes disturbances and is attended by a disruption of the physiological conditions as the cell cultures have to be temporarily removed from the incubator. Furthermore, the inherent inhomogeneity of the electric field across the cell layer typically leads to a systematic overestimation of TEER [13]. Altogether, there is a significant risk of obtaining erroneous results with this technical approach. 

### Automated impedance-based cell monitoring under physiological conditions using the cellZscope® device

#### Basics of the technique

**Figure 2:**
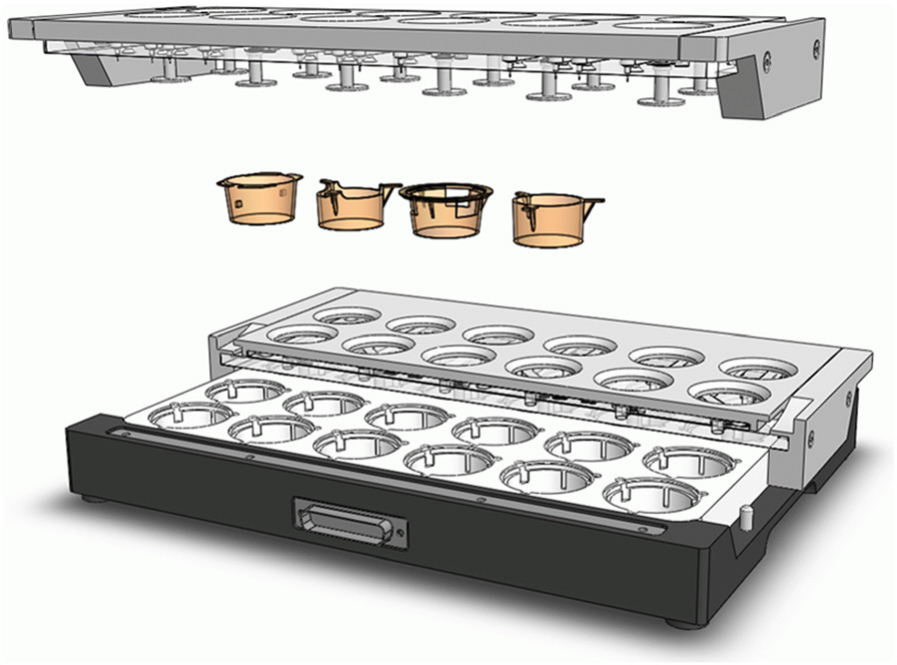
**Setup of the cellZscope device.** The cell module can be loaded with a broad range of standard cell culture inserts ranging from 6 to a maximum of 24 inserts. The TEER of all inserts can be measured continuously. During the experiment the cell module can be placed in a standard incubator. From [14] with permission.

Impedance spectroscopy is a highly reliable technique to measure the transendothelial or transepithelial electrical resistance (TEER). In impedance spectroscopy the frequency of an applied AC voltage is swept while measuring the amplitude and the phase of the resulting AC current. The resulting total impedance, Z, contains information not only about the TEER, but also the capacitance of the cell layer can be extracted and provided as a readout parameter. This non-invasive method can be applied to living cells without markers and allows them to be monitored during growth and differentiation [14]. Additionally, the barrier properties can be characterized before using the cultures in subsequent experiments. To examine the TEER of cells grown on the porous membrane of standard cell culture inserts, a computer-controlled multi-well device that automatically derives all important parameters has been developed (cellZscope, nanoAnalytics GmbH, Muenster, Germany, Figure 2). 

**Figure 3:**
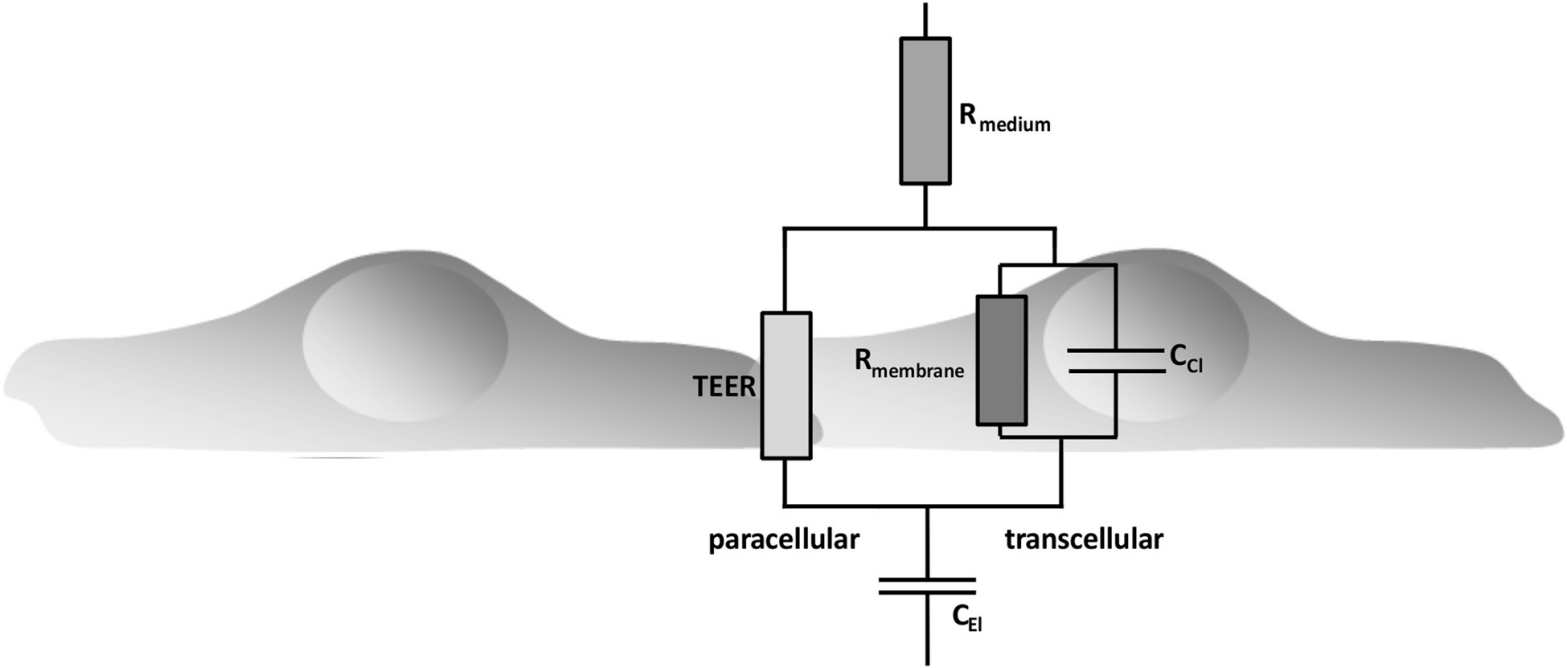
**Equivalent circuit diagram describing the contribution of the trans- and paracellular pathway to the total impedance, Z, of the cellular system.** TEER, transendothelial electric resistance; C_El_, capacitance of the electrodes; C_Cl_, capacitance of the cell layer; R_medium_, ohmic resistance of the medium; R_membrane_, ohmic resistance of the membranes. Please note that for most epithelial cells the TEER can be dominated by the transcellular pathway. This is true for tight epithelia already under resting conditions and, in leaky epithelia, after activation of ion channels.

**Figure 4:**
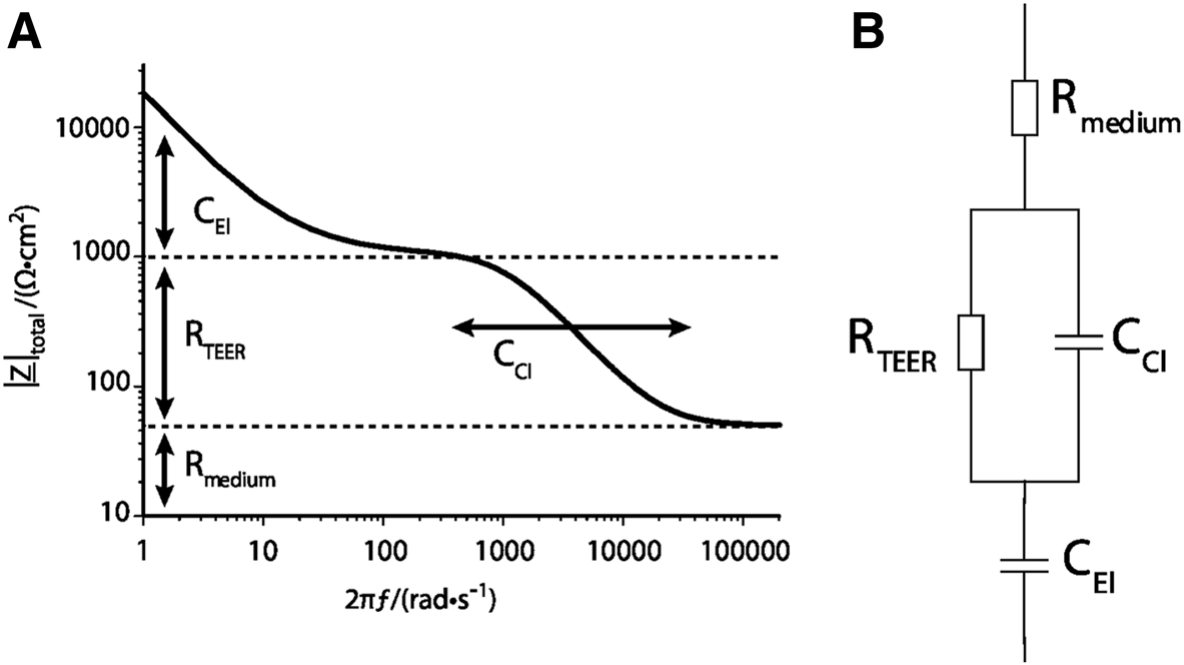
**(A) Schematic impedance spectrum of a cell monolayer at different frequencies.(B) Equivalent electrical circuit diagram for a cell monolayer.** At mid-range frequencies the cell-related parameters TEER and capacitance C_cl_ are contributing predominantly to the total impedance. At the lower end of the frequency range the spectrum is dominated by the capacity of the electrodes (C_El_). At high frequencies the capacitors C_cl_ and C_el_ become increasingly conductive and the remaining total impedance converges to the resistance of the medium (R_Medium_)_._ Adapted from [15] with permission.

In order to apply impedance spectroscopy to cellular systems and to retrieve the parameters needed to characterize barrier properties, an equivalent electrical circuit diagram and corresponding mathematical models have to be applied. A circuit diagram suitable to derive the total impedance Z of the cellular system is shown in Figure 3. Here, the current can either take the paracellular pathway through the intercellular cleft or the transcellular pathway across the cells. Within the paracellular pathway the tight junctional proteins represent an ohmic resistance (TEER) in the circuit diagram while each lipid bilayer in the transcellular pathway, can be described as a parallel circuit of an ohmic resistance (R_membrane_) and an electric capacitance, C_cl_. Within the considered frequency range, the high resistance of the membrane causes the current to flow predominantly across the capacitor and allows us, in first-order approximation, to ignore the membrane resistance and to summarize the apical and basolateral membranes in one capacitance (C_cl_). In addition, both the resistance of the surrounding medium (R_medium_) and the capacitance of the electrodes (C_el_) need to be considered as well. Taking all components together; a simplified electrical circuit diagram can be established and a non-linear frequency dependency of the total impedance, Z, is found. On the basis of the described parameters an algorithm can be used to fit the experimental data (Figure 4, [15]). At mid-range frequencies the cell-related parameters TEER and capacitance C_cl_ are predominantly contributing to the total impedance. At the lower end of the frequency range the spectrum is dominated by the capacity of the electrodes. At high frequencies the capacitors C_cl_ and C_el_ become increasingly conductive and the remaining total impedance converges to R_Medium_ (Figure 4). 

Based on the equivalent circuit, the corresponding modeling computer software can be employed to determine automatically best fit parameters and to extract the TEER and the capacitance C_cl_ as readout parameters. Further possible contributions to the cell layer`s total impedance (for example the cell membrane resistance change) can be neglected in this first order approximation. For a deeper understanding of the mathematical difference between resistance and impedance, imaginary numbers and vectors have to be considered [16].

### Applications of impedance measurements using filter systems

#### The effect of glucocorticoids on the properties of the blood-brain barrier

**Figure 5:**
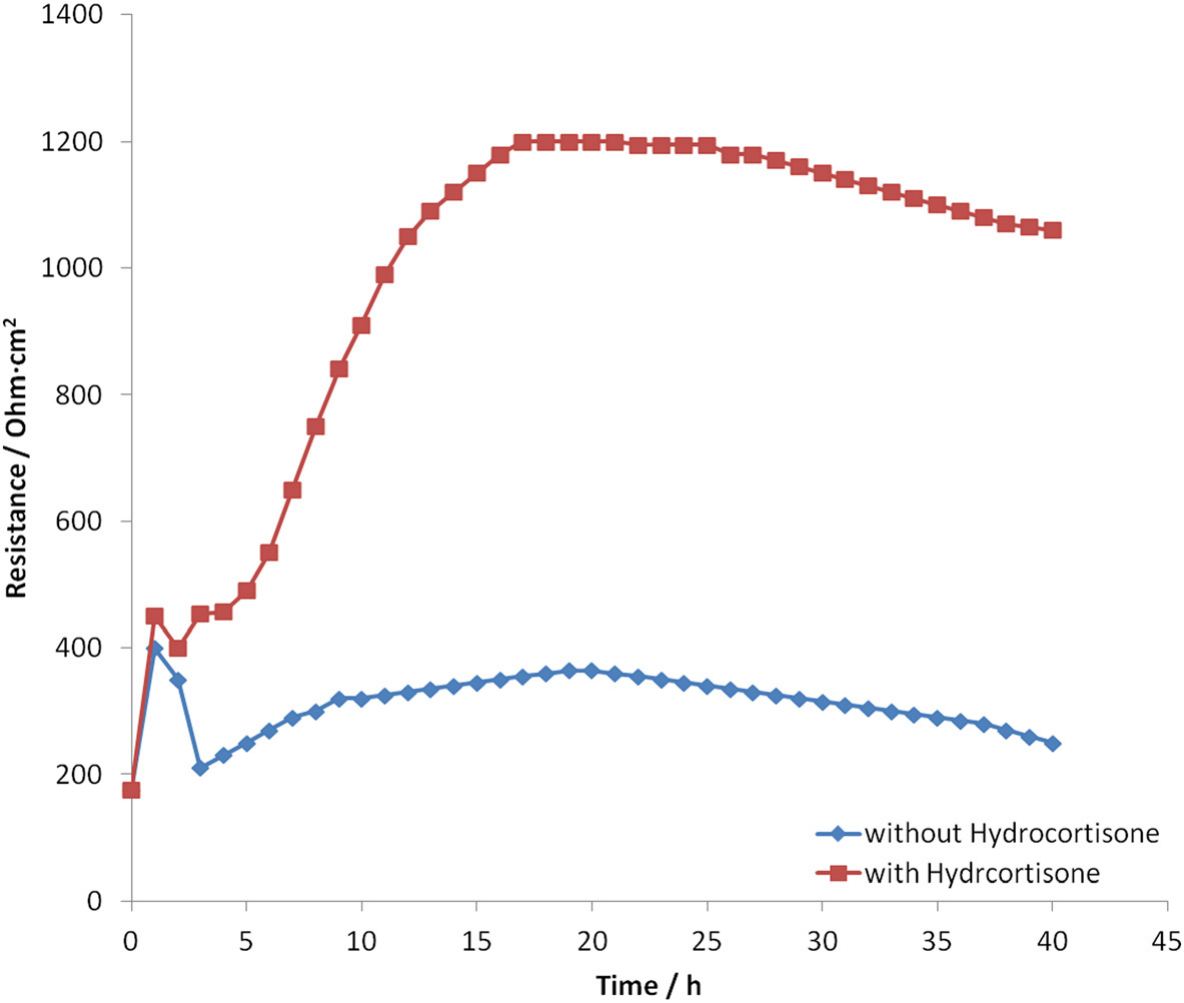
**Development over time of the TEER of primary porcine capillary endothelial cells cultured in serum-free medium supplemented with hydrocortisone (orange curve) and without hydrocortisone (blue curve):** In the presence of hydrocortisone an increase of the TEER is observed due to improved barrier integrity. Adapted from [18] with permission.

The barrier-strengthening effect of hydrocortisone has been proven by electrical measurements. Hoheisel *et al.*[17] were able to show an increase in TEER of porcine cells after addition of hydrocortisone (Figure 5) [18]. Also, Weidenfeller *et al.* were able to observe this barrier strengthening effect in murine cells after application of different glucocorticoids [19]. Using impedance measurements it was also found that the effect of glucocorticoids can be completely inhibited by the glucocorticoid-receptor antagonist mifepristone. Interestingly, the described effect can be achieved by a different range of glucocorticoids but not by mineralocorticoids. 

#### Impedance measurement as quality control

Techniques that quantify barrier properties have to achieve a good correlation with the permeability measurements of small, polar substances that are not substrates of BBB transporters. For impedance measurements, a constantly low sucrose permeability (value of p = 10^-7^ cm/s), which is characteristic for the BBB *in vivo,* was found in cells with TEER values > 600-800 Ω · cm^2^, while cells exhibiting a lower TEER showed a more variable permeability [11]. When performing transport experiments, it is of great importance to have an online control of barrier properties since a disruption of barrier integrity might result in false interpretation of data.

#### Nanoparticles at the blood–brain barrier7

**Figure 6:**
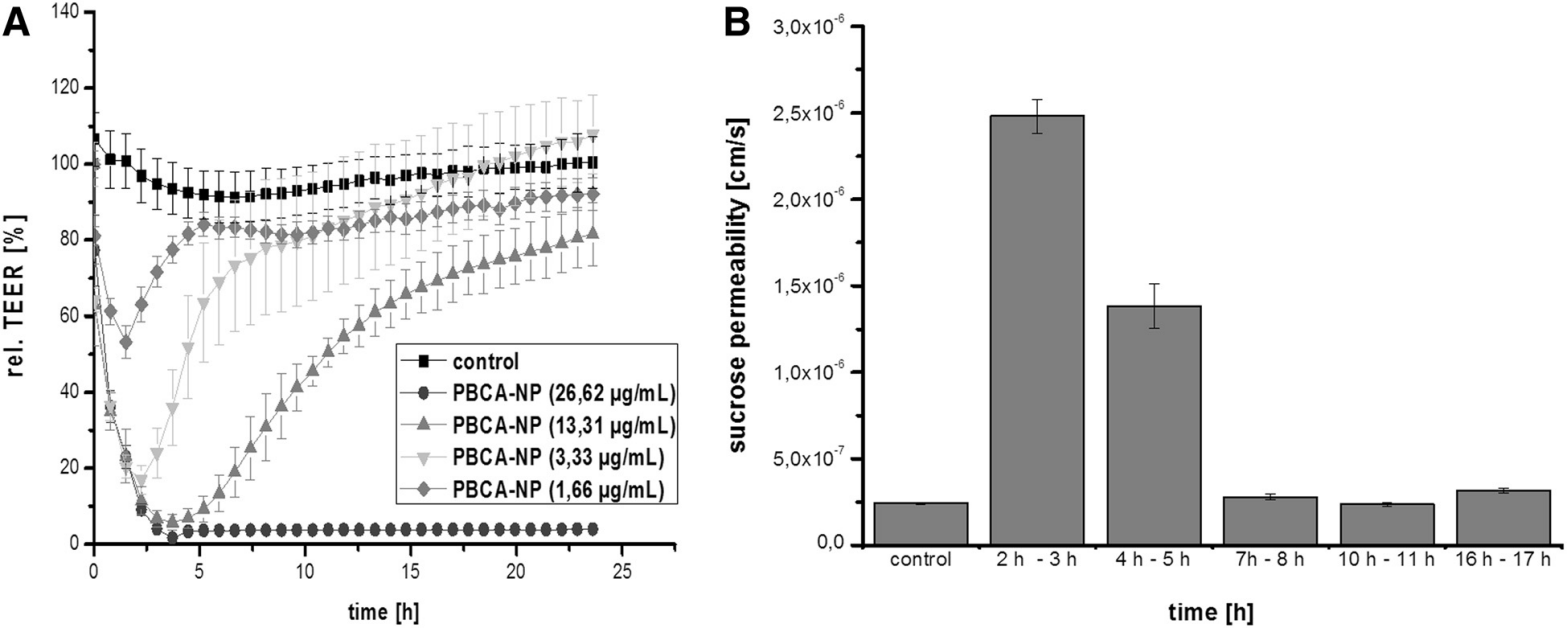
**Influence of poly(butyl)cyanoacrylate nanoparticles (PBCA-NP) on the integrity of porcine brain capillary endothelial cells (PBCEC).(A)** TEER development over time after the addition of PBCA-NP in different concentrations. **(B)**^14^C-Sucrose permeability at different times after the addition of PBCA-NP (13.31 μg/mL). From [20] with permission.

For several years, nanoparticles have been proposed as vehicles for drug delivery across the BBB. Recently, we have found that poly(butyl)cyanoacrylate nanoparticles (PBCA-NP) are able to temporarily open the blood–brain barrier [20]. After addition of PBCA-NP to porcine endothelial cells, a drastic decrease in barrier integrity was observed during 2 h, followed by a recovery of the barrier within the next 3 - 4 h (Figure 6). The observed reversible opening of the blood–brain barrier was also confirmed by sucrose and high molecular dextran permeability showing that the time-frame of barrier opening could also be employed to transfer a substance of interest from the blood-stream into the brain. 

Using impedance measurements, it is also possible to ascertain the toxicity of nanomaterials. Wagner *et al.*[21] have combined different oximes for treatment of organophosphate poisoning with human serum albumin nanoparticles to enable transport across the BBB. Using impedance spectroscopy, they were able to verify the presence of an intact barrier during their transport studies. Additionally, impedance measurements were used to assess the toxicity of the used nanoparticles underlining the results obtained from cytotoxicity assays.

#### Inflammatory cells cross the blood–brain barrier without opening the tight junctions

**Figure 7:**
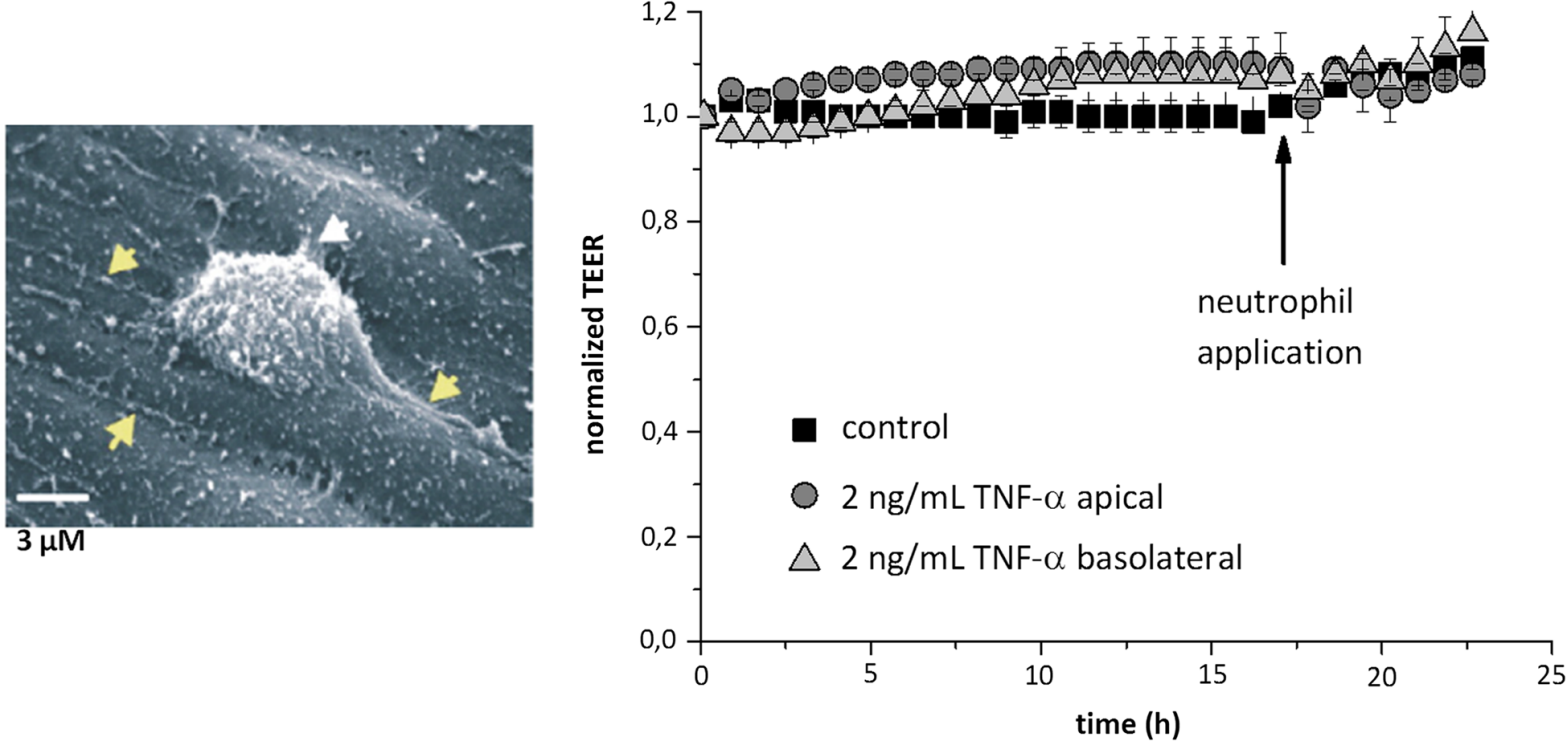
**The scanning electron microscope picture (left) shows a neutrophil that is invading the endothelial cell layer from the apical (blood) side.** The arrows on the right side show contact areas of the flattened neutrophil with the endothelial cell surface while the arrows on the left side point towards the tight junctions. Note the distance of the neutrophil to the cell junctions indicating transcellular migration. When monitored by TEER measurement (right) after inflammatory stimulation of the cells with TNF-α, neutrophil application did not result in a change in electrical resistance. Adapted from [22] with permission.

In several central nervous system diseases that involve BBB dysfunction an immense infiltration of the brain with leucocytes takes place. In general, two routes for their transmigration are being discussed. The transmigration could either take place via the paracellular route which would require a transient BBB breakdown, or via the transcellular pathway through the endothelial cell body. The latter route would probably not interfere with the tight junction organization and thereby leave the barrier intact. By an advanced microscopic *in vitro* study in a high-barrier porcine model, it has been shown that the diapedesis of leukocytes preferentially uses the transcellular route [22,23]. Here, a combination of impedance spectroscopy and advanced ultra-structural investigation demonstrated the lack of barrier impairment during the penetration process (Figure 7). 

#### Pericytes and astrocytes regulate the blood–brain barrier integrity

*In vivo,* the BBB properties are not solely due to the properties of capillary endothelial cells but are also induced by surrounding pericytes and astrocytes. The *in vitro* induction of BBB properties by astrocytes was characterized by determining the resistance in a co-culture-model of endothelial cells and astrocytes [24,25]. For all conditions tested, it was found that astrocytes increase BBB integrity. Also, the influence of pericytes on the integrity of the BBB is still under discussion. Employing TEER measurements, it was found that pericytes can either decrease or increase the integrity of the barrier, depending on their state of differentiation [26]. With the aid of resistance measurements, Nakagawa *et al.* were able to ascertain the best culture conditions for the establishment of a triple co-culture model of endothelial cells with astrocytes and pericytes [27]. The authors were able to show that the presence of astrocytes and pericytes resulted in an increase of TEER compared to the mono-culture.

### Electrical cell substrate impedance sensing

Electric cell-substrate impedance sensing (ECIS, Applied BioPhysics inc, Troy NY, USA) is an impedance-based method, which can be used as a tool for real-time monitoring of the cell behaviour such as adherence, mobility and growth on solid substrates [28]. This technique allows investigation of the invasive nature of cancer cells, the barrier function of endothelial cells, cell-cell and cell-matrix interactions, signal transduction for modern drug discovery and wound-healing processes.

In ECIS, cell layers are grown to confluence not on porous membranes but directly on integrated gold-film electrodes. The close proximity of the cell monolayer to the thin gold electrodes results in high sensitivity measurements. However, it is important to realize that there is no basolateral fluid compartment present due to the adherence of the cells to the electrode. This excludes the employment of the ECIS setup in transport or transfer experiments. There are also fundamental differences in the measured impedance data that need to be considered when comparing results obtained with membrane-based experimental setups such as the cellZscope [29]. This will be enlightened by some basic calculations in the following paragraph.

**Figure 8:**
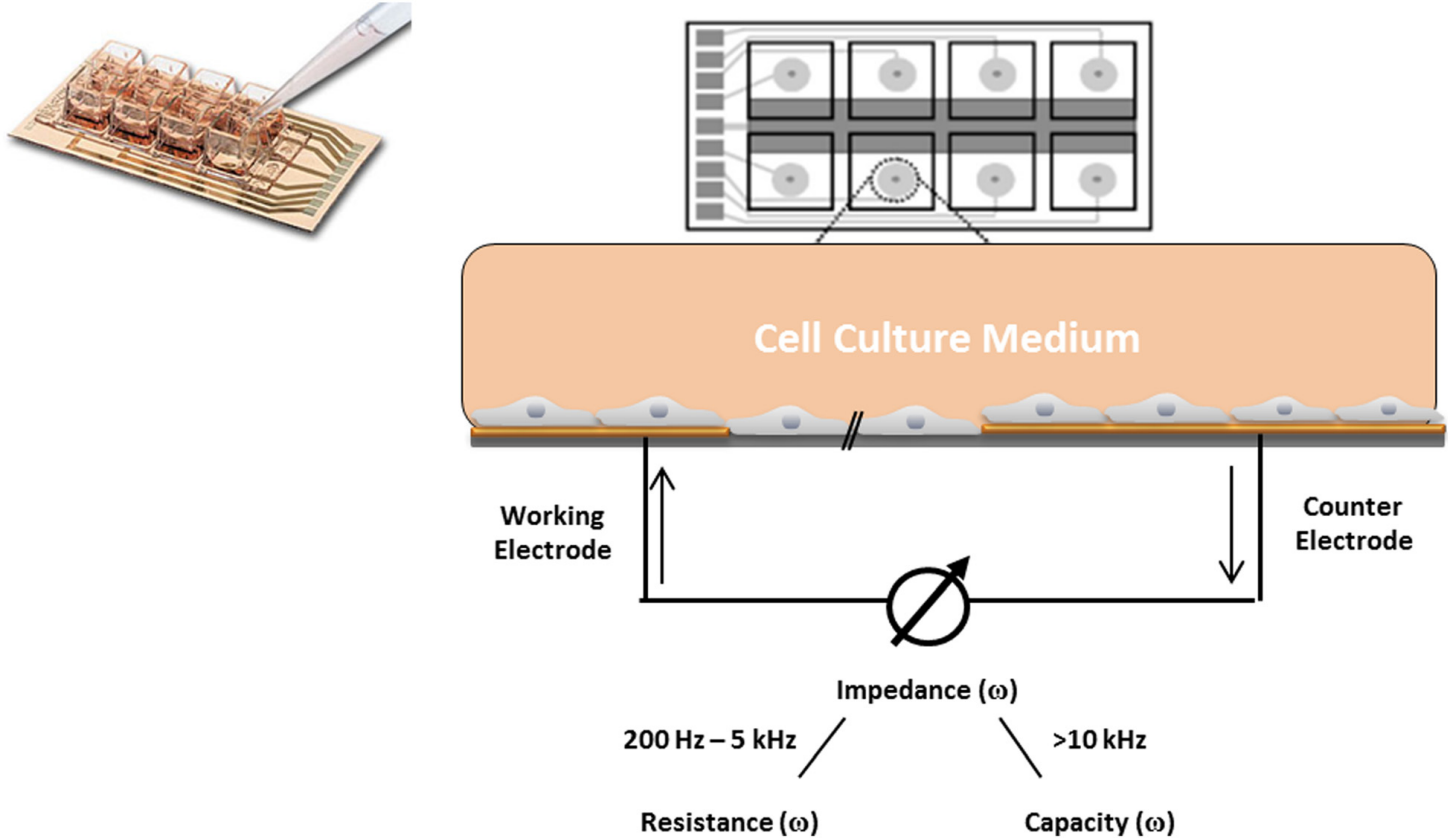
**Schematic drawing of an ECIS array and principle of the electric cell-substrate impedance sensing (ECIS) method.** Cell layers are grown to confluence on integrated gold-film electrodes. An applied AC current flows between small working electrodes and the larger counter electrode using normal culture medium as an electrolyte. By a variation of the frequency ω, a spectrum can be obtained. Applying higher frequencies the current flow is dominated by the capacity of the total system, at mid-range frequencies the ohmic resistance of the total system is mirrored. Adapted from Applied BioPhysics, Inc [28].

The ECIS array is typically composed of 8 wells which each contain 10 active working electrodes (diameter = 250 μm) connected in parallel (total area = 5×10^-3^ cm^2^, single electrode area = 5×10^-4^ cm^2^) and a larger in-plane counter electrode (area = 0.15 cm^2^), which are located at the base of the well. The core component of the arrays is the single impedance measurement chamber sketched in Figure 8. Because of the size differences in the electrodes, the measured electrical resistance is mainly determined by the working electrode. Due to the much smaller surface area of the active ECIS working electrodes (5×10^-3^ cm^2^) compared to a typical membrane insert and the corresponding electrode of about the same size (in the range of 0.3 to 4.7 cm^2^), the measured absolute impedance values differ significantly. If the ECIS device measures an absolute resistance of 1000 Ω at a given frequency, the results should be normalized by multiplying with the surface area of the working electrodes amounting to 5 Ω · cm^2^. This is a very low value typical for very leaky epithelia or endothelia, even though the measured value of 1000 Ω seems to be large at first glance. However, tight barriers should be expected to exhibit TEER values of 1000 Ω · cm^2^ or more, which corresponds to absolute resistance values of 200,000 Ω or more in an ECIS system. In case of the cellZscope the membrane area of the specific insert type is considered by the software and the TEER is automatically normalized and always given in Ω · cm^2^. This kind of normalisation to the active surface area is mandatory for comparing results obtained with different techniques.

**Figure 9:**
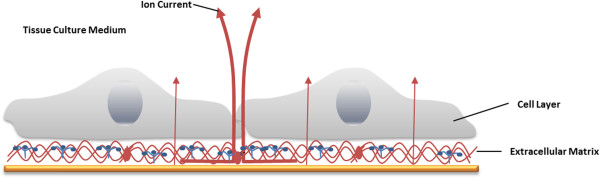
**The current pathway at low frequencies on a cerebral endothelial cell monolayer (ECIS method, 400 Hz).** At low frequencies the current predominantly flows paracellular (through extracellular matrix proteins) and between adjacent cells (through tight junctions) and the electrolyte (medium), see bold arrows. Adapted from Applied BioPhysics, Inc [28].

**Figure 10:**
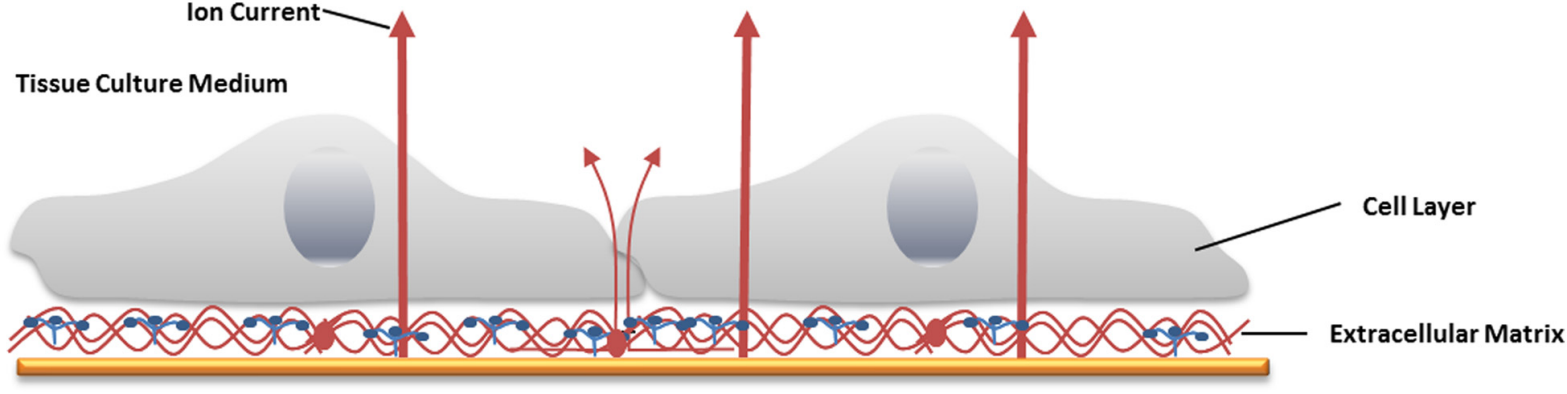
**By application of high frequencies (ECIS method, > 40 kHz), the capacitive amount of measured impedance is especially sensitive for adhered cells.** The current passes through the insulating cell monolayer, especially through cell membranes. Adapted from Applied BioPhysics, Inc [28].

An additional application of ECIS is to determine the electrical resistance of the cell-covered electrodes, as a function of AC frequency. At low frequencies, as cells attach and spread on the electrode surface, they act like insulting particles, forcing the current to flow around the cellular bodies on paracellular pathways. Thus, the ohmic resistance of the total system depends on the application of lower frequencies [30] (Figure 9). At high frequencies (f > 10 kHz) the majority of the current passes capacitatively across the basal and the apical cell membranes (transcellular pathway, Figure 10). Wegener *et al.* have proved that resistance measured at an AC frequency, f, of < 400 Hz is most sensitive for monitoring the establishment of cell-cell junctions as a function of time. Real-time monitoring and quantitative data for the adhesion process of cells on the electrodes can be measured at f > 40 kHz, which is a sensitive frequency range. Corresponding values for tight epithelia would be f < 10 Hz and f ~ 1000 Hz. Thereby, the progress of developing cell-cell contacts can be monitored during and after absorption of cells to the surface [30]. 

#### Cell attachment and cell growth

**Figure 11:**
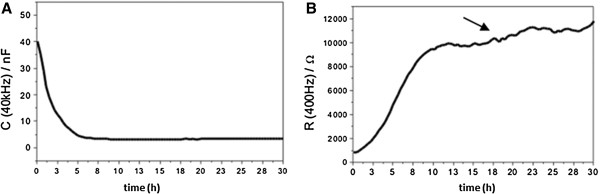
**Determination of the adhesion process and progression of the resistance of cells on ECIS arrays with time.(A)** The adhesion process can be determined by application of the frequency (f > 40 kHz). **(B)** By application of a frequency (f < 400 Hz) the development of cell-cell contacts (tight junctions) can be monitored. Bold arrow indicates the small fluctuations on the electrode due to micro motion in the cell monolayers. From [31] with permission.

ECIS allows researchers to monitor the attachment and spreading of mammalian cells in temporal resolution. Wegener *et al.* demonstrated that high frequency capacitance measurements (f = 40 kHz) are most suited to follow the increasing surface coverage of the electrodes by cells (Figure 11A, [31]). The impedance increases during the adherence to and spreading of cells over the electrode and and continues to increase within 10 hours, when porcine brain capillary endothelial cells (PBCEC) reach confluence (Figure 11B, [31]). The high sensitivity of the ECIS technique and its simultaneous high temporal resolution of one second, allows recording and analysis of the smallest cell movements and cell shape fluctuations in confluent monolayers, called micro motion [32]. The small fluctuations in the curves (Figure 11B, bold arrow) are due to micro motion of the cell monolayers on the electrode. 

**Figure 12:**
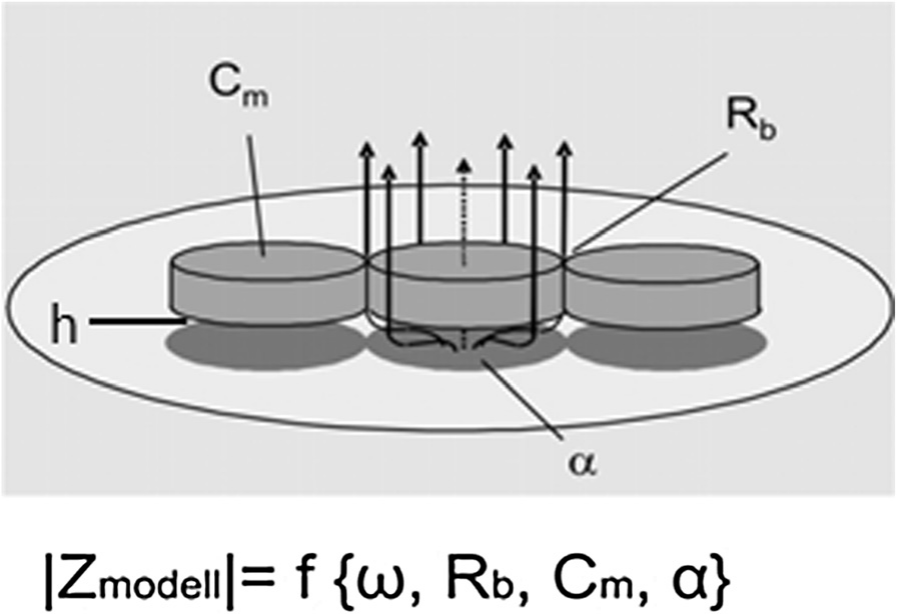
**Overview of the Giaever and Keese model **[32]**of the determination of specific parameters of cell-cell- and cell-substrate contact.** Cells are modeled as circular disks hovering at a distance *h* above the electrode surface. The solid lines indicate paracellular current flow, the broken line represents transcellular current flow. R_*b*_ represents resistance of the cell-cell contact; C_m_ explains the capacity of the cell layers and the *α* term describes the impedance contributions arising from the proximity of the cell monolayer to the surface of the electrodes (cell-substrate contacts). |Ζ_model_| describes the total impedance at different frequencies ω, the resistance of the cell-cell contact R_b_, capacitance C_m_ of the cell layers and the *α* term. For more details on the equation see reference [32]. From [31] with permission.

Besides the determination of the capacitance and impedance by using different frequencies, the analysis of the cell-cell contacts (R_b_), the capacitance of the cell layers (C_m_), and the close proximity of the cell monolayer to the surface of the electrodes, allows direct analysis of the amount of the impedance of the cell-matrix contacts (α). The cells are represented as cylinders, which keep a middle distance (h) to the surface of the gold electrodes (Figure 12) [32]. The cell-cell R_b_ and cell-substrate contacts, α, are serially connected and reflect the paracellular current flow. In parallel the transcellular current flows in a capacitive way through the cell membrane (C_m_). 

#### Extracellular matrix coating of ECIS electrodes

**Figure 13:**
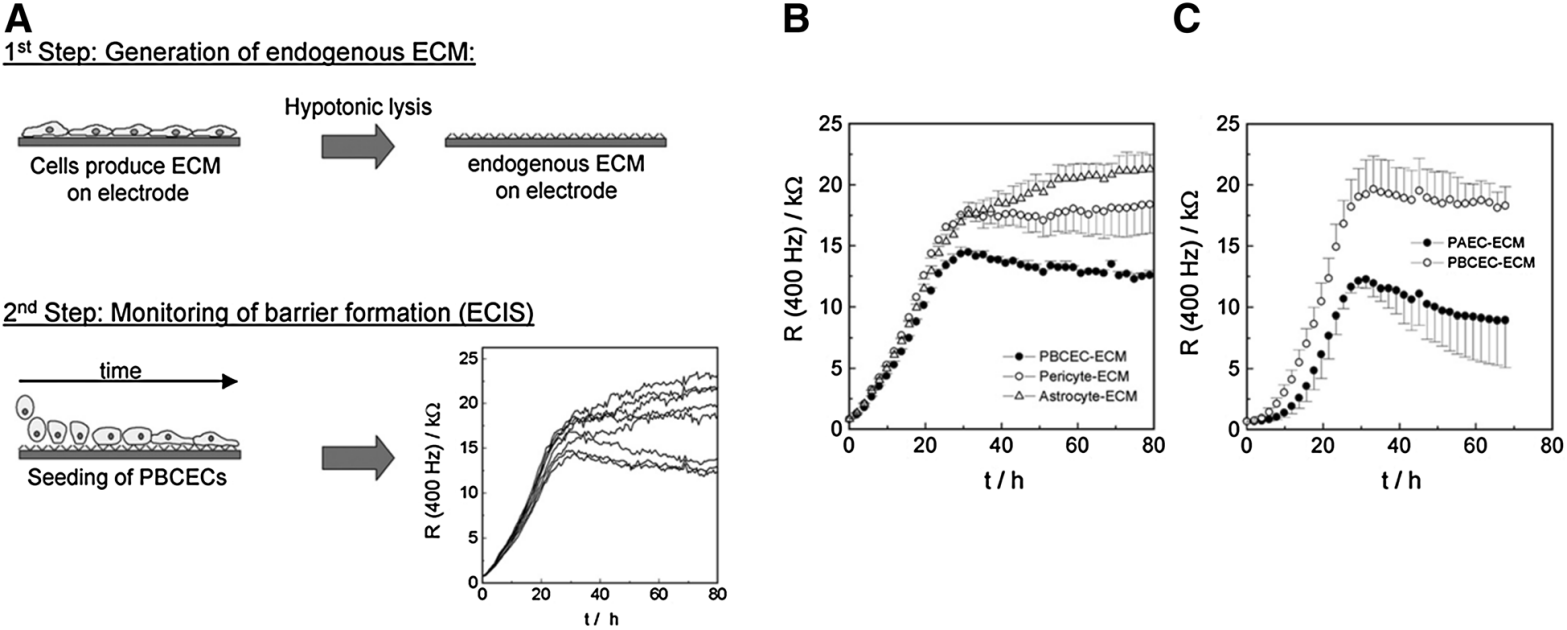
**Schematic illustration of the experimental setup to determine the impact of different endogenous extracellular matrices (ECM) on the integrity of brain capillary endothelial cells (PBCEC) using ECIS technique.(A)** Establishment of endogenous extracellular matrices derived from astrocytes, pericytes, aorta (PAEC) and cerebral endothelial cells (PBCEC) on the ECIS electrode surfaces (1^st^ step). Seeding of PBCEC on ECIS electrodes that had been pre-coated with endogenous extracellular matrices. Monitoring the progression of the barrier formation of PBCEC on different matrices (2^nd^ step) by ECIS technique. **(B,C)** Analysis of the impact of extracellular matrices on the barrier integrity on PBCECs. Time course of the resistance measured at a sampling frequency of 400 Hz. **(B)** Comparison between astrocyte, pericyte and PCBEC derived endogenous extracellular (as control) matrix on the integrity of PBCEC. **(C)** Represents the relation between aorta derived matrices to endothelial derived matrices (control). Each data point represents the mean ± SD From [33] with permission.

To clarify the impact of endogenous extracellular matrices on the barrier integrity of PBCEC, a generation model was established in two consecutive steps. The extracellular matrix (ECM)-donating cells (astrocytes, pericytes or endothelial cells) were grown to confluence and removed by a hypotonic lysis procedure from their ECMs (Figure 13A). Suspensions of PBCEC were seeded on the remaining matrices and the formation of the BBB was monitored at a frequency of R (400 Hz/kΩ) over a defined period of time (Figure 13B; C). The authors observed an improved integrity of the BBB by pericyte- and astrocyte-derived ECM compared to endothelial cell-derived ECM alone, which served as a control (B). Non-brain capillary aorta endothelial cell-derived ECM markedly reduced the electrical resistance of PBCEC (C) [33]. 

#### Wound healing

**Figure 14:**
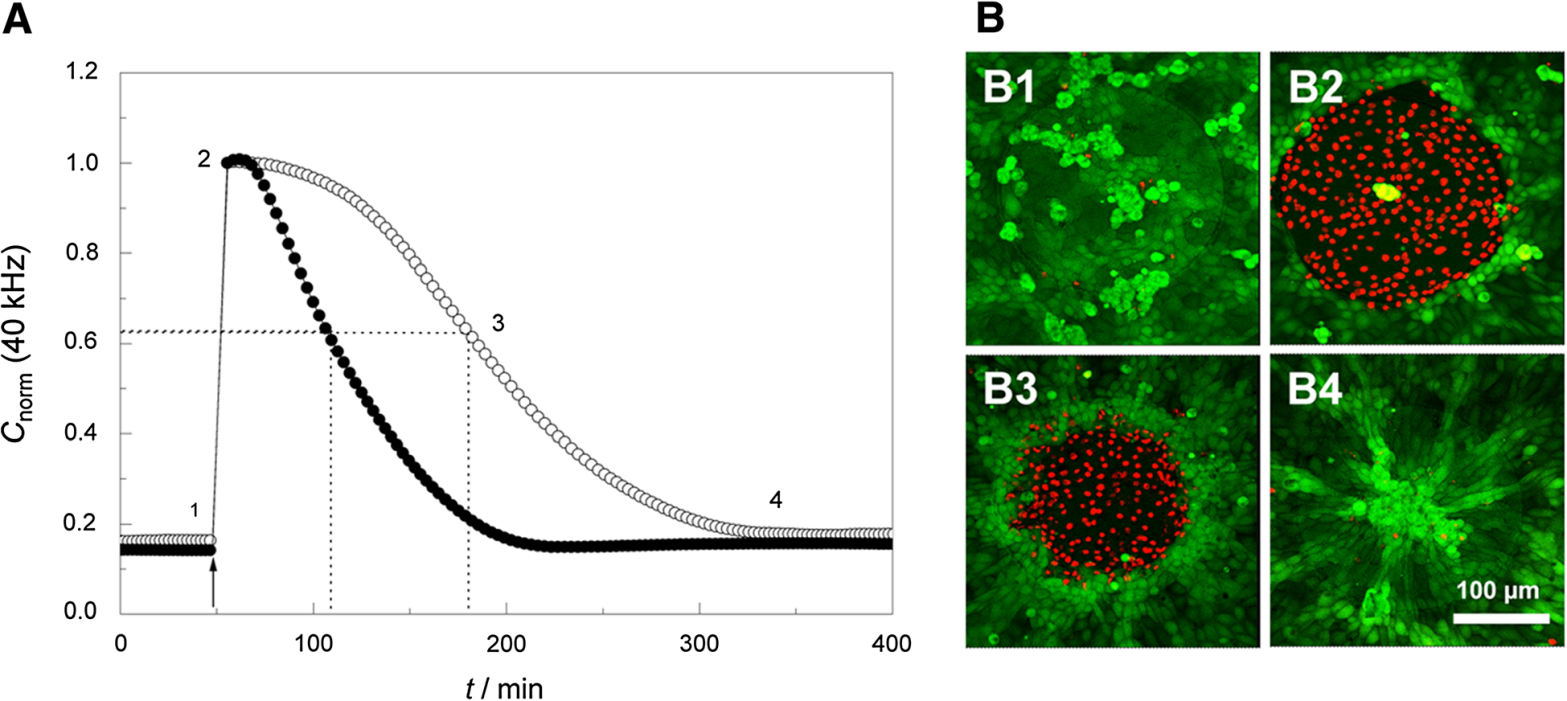
**(A) Time course of normalized capacitance C of a sampling frequency of 40 kHz wounding of normal rat kidney (NRK) epithelial cells (wounding parameters: 4 V, 20 s, 40 kHz).** The arrow indicates the time point of injury (2). **(B)** Confocal laser scanning microscopy (CLSM) images of the Live/Dead Assay show vital cells surrounding the active electrode surface (calcein acetoxymethylester stained in green) and dead cells (ethidium homodimer-1 stained in red) on the electrode itself. (B; 1-4) Documentation of the wound healing process by CLSM images, 1 = before wounding, 2 = after wounding, 3 = after partial wound healing, 4 = after complete wound healing. From [34] with permission.

In a wound healing-assay, cell monolayers on ECIS electrodes were subjected to an insulting current, resulting in severe electroporation and subsequent cell death. The authors were able to monitor the ultimate healing after electrical wounding of normal rat kidney (NRK) epithelial cells over a period of time (Figure 14A) and to determine the time points of the ingrowth of cells from the periphery to the electrodes by using confocal laser scanning microscopy (CLSM) (Figure 14B) [34]. Besides finding a suitable frequency for wounding, the amplitude and the pulse duration of the wounding pulse had to be optimized as well. Wounding parameters for NRK cells were 4 V, 20 s, 40 kHz. 

### Two-path impedance spectroscopy

So far we have only considered changes in the paracellular resistance and the sub-epithelial resistance caused by the cell attachment. However, solutes may also be transported via a transcellular way crossing the apical and the basolateral membrane. Thus, it might become desirable to determine the para- and the transcellular resistance separately. This is especially important if ion fluxes via ion channels are involved. Krug *et al.* have developed a technique based on impedance spectroscopy which enables them to differentiate between the transcellular and paracellular pathways. In combination with flux measurements using e.g. fluorescein as a paracellular marker they are able to determine the transcellular resistance as well [35].

## Conclusions

Electrical resistance measurements are valuable tools to quantify barrier properties. Impedance spectroscopy is a new non-invasive technique to monitor not only barrier function but also processes, like cell growth and cell differentiation. The main advantage of impedance spectroscopy is the automated monitoring process. Two main systems have to be distinguished. One is the cellZscope device, using standard cell culture inserts with semi-permeable membranes as substrates for cell growth, thus allowing simultaneous transport investigations. The second is the ECIS system, where the cells are directly grown on the electrode thus losing their basolateral compartment. However, in addition the value α which characterises the cell-matrix interaction, can be determined. Moreover, since higher currents might be applied locally, cells can be locally destroyed to allow measurements of wound healing. Thus, both experimental methods are important, their use depending on the scientific question under investigation.

## Abbreviations

α: Impedence Of Cell-Matrix Contacts; AC: Alternating Current; BBB: Blood–brain Barrier; C_Cl_: Capacitance Of The Cell Membrane; C_El_: Capacitance Of The Electrodes; C_m_: Capacitance Of The Membrane In ECIS; CLSM: Confocal Laser Scanning Microscopy; CSF: Cerebrospinal Fluid; DC: Direct Current; ECIS: Electrical Cell Substrate Impedance Sensing; ECM: Extracellular Matrix; EVOM: Epithelial Voltohmmeter; F: Frequency; I: Current; NRK: Normal Rat Kidney; PBCA-NP: Poly(Butyl)Cyanoacrylate - Nanoparticles; PBCEC: Porcine Brain Capillary Endothelial Cells; R: Ohmic Resistance; R_b_: Ohmic Resistance Of Cell-Cell Contacts; R_membrane_: Ohmic Resistance Of The Membrane; TEER: Transendothelial Electrical Resistance; TNF-α: Tumor Necrosis Factor - α; U: Voltage; Z: Impedance.

## Competing interests

Prof. Galla is a scientific advisor of the nanoAnalytics GmbH, manufacturer of cellZscope®.

## Authors’ contributions

All authors contributed equally. All authors have read and approved the final version of the manuscript.
